# Perceived health, psychological distress and quality of life in 8415 adults with congenital heart disease from 32 countries

**DOI:** 10.1136/heartjnl-2024-325296

**Published:** 2025-06-27

**Authors:** Philip Moons, Adrienne H Kovacs, Eva Goossens, Koen Luyckx, Laila Ladak, Mohamed Leye, Alexander Van De Bruaene, Ming Chern Leong, Anna Kaneva, Paulo Henrique Manso, John Jairo Araujo, Navaneetha Sasikumar, Harald Gabriel, Dejuma Yadeta, Jou-Kou Wang, Junko Enomoto, Maria Emilia Areias, Diamantis Kosmidis, Louise Coats, Anne Marie Valente, Ju Ryoung Moon, Magalie Ladouceur, Corina Thomet, Jamie L Jackson, Camilla Sandberg, Edward Callus, Yuli Y Kim, Birgitte Lykkeberg, Luis Alday, Charlène Bredy, Arwa Saidi, Fernando Baraona Reyes, Samuel Menahem, Michèle de Hosson, Joanna Hlebowicz, Christina Christersson, Ali N Zaidi, Bengt Johansson, Brith Andresen, Jean-Claude Ambassa, Eva Mattsson, Andrew Constantine, Pascal Amedro, Joost P van Melle, Shelby Kutty, Lucia Ortiz, Fatma Demir, Paul Khairy, Jonathan Windram, Judith Bouchardy, Maryanne Caruana, Susan M Jameson, Vaikom S Mahadevan, Lidija B McGrath, Julius Chacha Mwita, Liesbet Van Bulck, the APPROACH-IS II consortium, on behalf of the International Society for Adults Congenital Heart Disease (ISACHD), Navaneetha Sasikumar

**Affiliations:** 1 University of Gothenburg, Gothenburg, Sweden; 2 KU Leuven, Leuven, Belgium; 3 University of Cape Town, Cape Town, South Africa; 4 Equilibria Psychological Health, Toronto, Ontario, Canada; 5 University of Antwerp, Antwerpen, Belgium; 6 University of the Free State, Bloemfontein, South Africa; 7 The Aga Khan University, Karachi, Pakistan; 8 University of Thies Faculty of Health Sciences, Thies, Senegal; 9 Structural and Congenital Cardiology, University Hospitals Leuven, Leuven, Belgium; 10 Paediatric & Congenital Heart Centre, Institut Jantung Negara, Kuala Lumpur, Malaysia; 11 National Heart Hospital, Sofia, Bulgaria; 12 University Hospital, Ribeirão Preto, Brazil; 13 Meintegral-Clinic, Manizales, Colombia; 14 Amrita Institute of Medical Sciences, Kochi, Kerala, India; 15 Medical University of Vienna, Vienna, Austria; 16 Internal Medicine, School of Medicine, Addis Ababa University, Addis Ababa, Eritrea; 17 Pediatrics, National Taiwan University Hospital, Taipei, Taiwan; 18 Toyo University, Tokyo, Japan; 19 Chiba Cerebral and Cardiovascular Center, Chiba, Japan; 20 University of Porto, Porto, Portugal; 21 Centro Hospitalar Universitário de S. João, Porto, Portugal; 22 University General Hospital of Thessaloniki AHEPA First Cardiology Clinic, Thessaloniki, Greece; 23 Newcastle University, Newcastle upon Tyne, UK; 24 Cardiology, Children's Hospital Boston and Women's Hospital, Boston, Massachusetts, USA; 25 Samsung Medical Center, Gangnam-gu, Seoul, Republic of Korea; 26 Cardiology, Hopital Europeen Georges Pompidou, Paris, France; 27 HUG, Geneva, Switzerland; 28 Inselspital University Hospital Bern, Bern, Switzerland; 29 Center for Biobehavioral Health, Nationwide Children’s Hospital, Columbus, Ohio, USA; 30 Department of Public Health and Clinical Medicine, Umeå University, Umeå, Sweden; 31 IRCCS Policlinico San Donato, San Donato Milanese, Lombardia, Italy; 32 University of Milan, Milan, Italy; 33 Philadelphia Adult Congenital Heart Center, Penn Medicine and Children’s Hospital of Philadelphia, Philadelphia, Pennsylvania, USA; 34 Copenhagen University Hospital, Kobenhavn, Denmark; 35 Hospital de Niños, Cordoba, Argentina; 36 Pediatric Cardiology, Centre Hospitalier Regional Universitaire de Montpellier, Montpellier, France; 37 Cardiology, University of Florida, Gainesville, Florida, USA; 38 Pontificia Universidad Católica de Chile, Santiago, Chile; 39 Department of Paediatrics, Monash University, Melbourne, Victoria, Australia; 40 Department of Adult Congenital Cardiology, University Hospital Ghent, Gent, Belgium; 41 Lund University, Skåne University Hospital, Lund, Sweden; 42 Cardiology, Medical Sciences, Uppsala, Sweden; 43 Cardiology, Mount Sinai School of Medicine, New York, New York, USA; 44 Oslo Universitetssykehus Intervensjonssenteret, Oslo, Norway; 45 Clinique Medical Le Jourdain, Yaoundé, Cameroon; 46 Karolinska Institute, Stockholm, Sweden; 47 Royal Brompton and Harefield Hospitals, London, UK; 48 Pediatric and Congenital Cardiology, CHU de Bordeaux Hôpital Cardiologique, Pessac, France; 49 University Medical Centre Groningen, Groningen, The Netherlands; 50 Johns Hopkins University, Baltimore, Maryland, USA; 51 Hospital San Juan De Dios De La Plata, Buenos Aires, Argentina; 52 Ege University Institute of Health Sciences, Izmir, Turkey; 53 Univ Montreal, Montreal, Quebec, Canada; 54 Mazankowski Alberta Heart Institute, Edmonton, Alberta, Canada; 55 Department of Cardiology and Cardiac Surgery, Lausanne University Hospital, Lausanne, Switzerland; 56 Cardiology, Mater Dei Hospital, Msida, Malta; 57 Lucile Packard Children’s Hospital School, Palo Alto, California, USA; 58 Department of Cardiology, University of California San Francisco, San Francisco, California, USA; 59 University of Massachusetts Chan School of Medicine, Worcester, Massachusetts, USA; 60 Oregon Health & Science University, Portland, Oregon, USA; 61 Internal Medicine, University of Botswana, Gaborone, Botswana; 62 Research Foundation Flanders, FWO, Brussels, Belgium

**Keywords:** Heart Defects, Congenital, Health Services, Global Health

## Abstract

**ABSTRACT:**

**Background:**

The global prevalence of congenital heart disease (CHD) is increasing. Research on patient-reported outcomes (PROs) predominantly originates from high-income countries, resulting in an incomplete understanding of the true global burden of CHD from the patient perspective. Therefore, we described perceived health, psychological distress and quality of life (QoL) in a large sample of adults with CHD from the globe and explored the relationship between PROs and the income level of the countries.

**Methods:**

Assessment of Patterns of Patient-Reported Outcomes in Adults with Congenital Heart Disease—International Study II (APPROACH-IS II) represents an international cross-sectional investigation of PROs in 8415 patients from 53 centres across 32 countries. Patients completed questionnaires to measure perceived health status (RAND-12 Health Survey; EuroQOL-5D Visual Analog Scale); depressive symptoms (Patient Health Questionnaire-8, PHQ-8); anxiety (Generalized Anxiety Disorder Scale-7) and QoL (Linear Analog Scale). Gross National Income per capita in US dollars was used for stratifying countries according to income levels.

**Results:**

Large intercountry disparities in PROs were observed. Switzerland demonstrated the highest mean scores for physical functioning, self-rated health and QoL, while Senegal had the lowest scores. Patients from Malta demonstrated the highest mean scores for mental health, and Senegal had the lowest scores. With regard to depressive symptoms and anxiety, Pakistan had the lowest mean scores, while Turkey had the highest scores. Patients from high-income nations reported significantly better physical functioning, mental functioning and QoL.

**Conclusion:**

Large intercountry disparities in PROs were observed. APPROACH-IS II is a pioneering international endeavour that comprehensively evaluated PROs among adults with CHD, drawing participants from nations with different income levels.

**Trial registration number:**

NCT04902768.

**WHAT IS ALREADY KNOWN ON THIS TOPIC**

- Research on patient-reported outcomes (PROs) in congenital heart disease (CHD) predominantly originates from high-income countries.
- Prior research in high-income countries demonstrated intercountry variation in PROs, although the explained variance of country level was limited.

**WHAT THIS STUDY ADDS**

- Here, we investigated PROs in adults with CHD from 32 countries, including also countries with a lower income level.
- Large intercountry disparities in PROs were observed.
- Self-reported physical functioning, mental health and quality of life were positively associated to the income level of the countries.

**HOW THIS STUDY MIGHT AFFECT RESEARCH, PRACTICE OR POLICY**

- The present study gives a better view on the global burden of CHD from the patient perspective.

## Introduction

 Congenital heart disease (CHD) is the most common birth defect, with a global birth prevalence of 9.4 per 1000.^1^ Prior epidemiological studies have shown geographic variations.^1^ There are also substantial variations and disparities worldwide with respect to outcomes. The Global Burden of Disease Study indicated that mortality in people with CHD is predictably highest in countries with the worst socioeconomic status.^2^ With temporal improvements in survival,^3^ the epidemiology and consequently the burden of CHD worldwide is changing. Globally, and even more so in low and middle-income countries, the contribution of CHD as a cause of death is increasing.^2^ This means that interventions and resources are increasingly directed to improve survival and optimise patients’ quality of life (QoL).^2^

QoL is a well-established patient-reported outcome (PRO). PROs are defined as: *any report of the status of a patient’s health condition, health behaviour, or experience with healthcare that comes directly from the patient, without interpretation of the patient’s response by a clinician or anyone else*.^4^ PROs are increasingly recognised as essential in understanding patients’ perspectives of their health and medical condition, as they provide valuable information to clinicians beyond traditional outcomes such as mortality and morbidity.^5^ However, research on PROs is primarily conducted in high-income countries, which hampers the generalisability and relevance to the global population of affected patients. Consequently, the true burden of CHD around the globe as perceived by patients is unknown.

From 2013 to 2015, the ‘Assessment of Patterns of Patient-Reported Outcomes in Adults with Congenital Heart Disease—International Study’ (APPROACH-IS) was conducted in 15 countries.^6 7^ Thirteen of these countries were high-income countries and two were middle-income countries.^8^ Expanding such a study to low and lower middle-income countries was paramount to gain a global view of the burden of CHD. Therefore, the aims of this study were (1) to describe PROs in a large sample of adults with CHD from around the globe and (2) to explore the relationship between PROs and the income level of high, upper middle, lower middle and low-income countries.

## Methods

We established the APPROACH-IS II consortium that included 53 centres from 32 countries across six continents.^9^ Of the participating countries, 20 were high income, 7 upper middle income; 4 lower middle-income and 1 low-income country. APPROACH-IS II used a cross-sectional design. Approval of the main study protocol was obtained from the Institutional Review Board of the University Hospitals Leuven/KU Leuven, that is the coordinating centre, while each participating centre obtained local ethics approval for study execution. All participants were required to provide written informed consent, although legislation in certain regions exempted survey studies from this requirement. The project was conducted in accordance with the declaration of Helsinki. The protocol is registered at ClinicalTrials.gov (NCT04902768), and detailed information on the rationale, design and methods of APPROACH-IS II is available in a published methods paper.^9^

### Study population and procedure

Inclusion criteria were: (1) patients with diagnosis of CHD, defined as: *a gross structural abnormality of the heart and/or intra-thoracic great vessels that is actually or potentially of functional significance (including mild, moderate and complex heart defects*)^10^; (2) aged 18 years or older at the date of study entry; (3) diagnosis of CHD before the age of 10 years; (4) followed at an adult CHD centre or included in a national/regional registry; (5) demonstrated physical, cognitive and language abilities required to complete self-report questionnaires. Patients who had received a heart transplant before study participation were excluded. Data collection ran from August 2019 to December 2022. From March 2020 until June 2020, data collection was paused in all centres to avoid a potential bias in PROs due to the initial lockdowns in the COVID-19 pandemic.^11^ After that period, data collection was resumed depending on the local situation and subsequent lockdowns, which differed across the globe.

A total of 8415 adults with CHD were enrolled in APPROACH-IS II. Patients completed PRO measures at home (n=3939; 47.0%) or onsite (n=4006; 47.8%); 1753 patients (20.9%) completed the surveys online (via REDCap)^12^ and 6192 patients (73.9%) completed pen and paper surveys. In France, Portugal, Pakistan and Senegal, some patients (n=433; 5.2%) were interviewed by telephone. Data collected on paper or via phone were entered into REDCap by a data collection officer.

### Measures

In order to capture the outcomes that matter most to patients, and to integrate their perspective in the design of this study, we relied on the standard set of PROs for CHD as recommended by the International Consortium for Health Outcomes Measurement (ICHOM), in which patients were involved. The following PRO measures were administered: the 12-item shortened and adapted version of the RAND-36^13^ and the EuroQOL-5D Visual Analog Scale^14^ to assess perceived health status; the PHQ-8 for depressive symptoms^15^; the General Anxiety Disorder-7 for anxiety symptoms^16^ and a Linear Analog Scale for QoL.^17^ Online supplemental table S2 provides an expanded definition of the domains as applied in APPROACH-IS II as well as the interpretation of scores for the individual questionnaires. Details on the instruments and their psychometric properties are available in the published APPROACH-IS II methods paper.^9^ Medical data were collected from the health records of each study participant.

For each participating country, the Gross National Income (GNI) per capita in US dollars, converted from local currency using the World Bank Atlas method was used (https://data.worldbank.org/indicator/NY.GNP.PCAP.CD). This GNI is used by the World Bank to categorise countries into high (GNI≥US$ 13 846), upper-middle (GNI US$ 4466–13 845), lower-middle (GNI US$ 1136–4465) and low income (GNI ≤US$ 1135) (data in 2022: https://www.worldbank.org/en/country/mic/overview).

### Statistical analysis

PRO data were presented as means and SD, and categorical variables were rendered as absolute numbers and proportions. Missing values for the PROs ranged from 0.06% to 2%. The Little Missing Completely at Random test was non-significant, indicating that there is no evidence that missingness is associated with the values of the other outcomes. Therefore, missing data for the PROs were imputed using the single-imputation expectation-maximisation method.

The relationships of country income with PROs were investigated using multivariable general linear mixed models. The data followed a two-level structure, with patients nested within their respective centres. Hence, centre was used as random effect. To deal with the non-linear relationship between GNI and the PROs, a Log2 transformation was applied on the GNI. IBM SPSS Statistics for Windows, V.29 (Armonk, New York: IBM), was used for data analysis. Additionally, Ridgeline plots were created using RStudio, V.1.1.463, to visually represent the data distribution. A significance level of p<0.05 was employed as the threshold for statistical significance, and all statistical tests were two sided. Effect sizes were assessed using Cohen’s d, for which the following cut-off values were used: 0.2 to 0.5, indicative of a small effect; 0.5 to 0.8, a moderate effect; and >0.8, a large effect.^18^

## Results

### Sample characteristics

Characteristics of the 8415 participants are detailed in table 1. Patients had a median age of 32 years and 53.9% were women. Most participants had a white or Caucasian background, held a high school diploma, either worked part time or full time and were married or cohabitating with a partner. With regards to their medical profile, 58.2% had CHD of moderate complexity, 52.1% were in physiological stage C, and 67.5% were in New York Heart Association functional class I, indicating that they were asymptomatic. The heart defects most represented were repaired tetralogy of Fallot, transposition of the great arteries, congenital aortic valve disease and coarctation of the aorta. In this sample, 74.2% of the patients had undergone prior cardiac surgery and 33.6% received catheter interventions (table 1). A detailed description of patient characteristics per country is provided in online supplemental table S1.

**Table 1:** Demographic and medical background variables for the total sample of adults with congenital heart disease (n=8415)

Variables	n (%)
Sex (n=8393)	
Men	3864 (46.0)
Women	4522 (53.9)
Other	7 (0.1)
Median age in years (n=8392)	32.0 (IQR 25–43)
Background (n=8402)	
White or Caucasian	5578 (66.4)
Asian	1337 (15.9)
Hispanic or Latino	700 (8.3)
Black or African-American	451 (5.4)
Middle Eastern or Arabic	92 (1.1)
Other	244 (2.9)
Educational level (n=8306)	
Less than high school	1241 (14.9)
High school	3492 (42.0)
Bachelor or college degree	2353 (28.3)
Master’s degree or higher	1220 (14.7)
Employment status (n=8359)	
Part-time or full-time work	4854 (58.1)
Job seeking, unemployed or disability	1267 (15.2)
Homemaker or retired	836 (10.0)
Full-time student	787 (9.4)
Other	615 (7.4)
Marital status (n=8376)	
Married or living with partner	4055 (48.4)
Never married	3877 (46.3)
Divorced or widowed	418 (5.0)
Other	25 (0.3)
Anatomical complexity of ACHD AP (n=8130)	
Simple	1187 (14.6)
Moderate	4740 (58.3)
Complex	2203 (27.1)
Physiological stage of ACHD AP (n=8008)	
Stage A	888 (11.1)
Stage B	2375 (29.7)
Stage C	4166 (52.0)
Stage D	579 (7.2)
New York Heart Association assessment (n=8057)	
Class I	5433 (67.4)
Class II	2141 (26.6)
Class III	453 (5.6)
Class IV	30 (0.4)
Heart defect (n=8130)	
Repaired tetralogy of Fallot	1334 (16.4)
TGA (d-TGA or CCTGA)	897 (11.0)
Congenital aortic valve disease	791 (9.7)
Coarctation of the aorta	729 (9.0)
Fontan procedure	441 (5.4)
Repaired secundum ASD or sinus venosus defect without significant residual shunt or chamber enlargement	355 (4.4)
Pulmonary valve stenosis (moderate or greater)	311 (3.8)
Isolated small VSD	285 (3.5)
VSD with associated abnormality and/or moderate or greater shunt	262 (3.2)
AVSD (partial or complete, including primum ASD)	251 (3.1)
Repaired VSD without significant residual shunt or chamber enlargement	229 (2.8)
Single ventricle (including double inlet left ventricle, tricuspid atresia, hypoplastic left heart, any other anatomic abnormality with a functionally single ventricle)	222 (2.7)
Pulmonary atresia (all forms)	212 (2.6)
Ebstein anomaly (disease spectrum includes mild, moderate and severe variations)	199 (2.4)
Double-outlet ventricle	153 (1.9)
Congenital mitral valve disease	137 (1.7)
Moderate and large unrepaired secundum ASD	115 (1.4)
Anomalous pulmonary venous connection, partial or total	109 (1.3)
Cyanotic defect (unrepaired or palliated, all forms) for example, unrepaired tetralogy of Fallot	100 (1.2)
Subvalvar aortic stenosis (excluding HCM)	94 (1.2)
Isolated small ASD	91 (1.1)
Truncus arteriosus	74 (0.9)
Mild isolated pulmonic stenosis	57 (0.7)
Pulmonary valve regurgitation (moderate or greater)	50 (0.6)
Previously ligated or occluded ductus arteriosus	46 (0.6)
Moderate and large persistently patent ductus arteriosus	41 (0.5)
Supravalvar aortic stenosis	35 (0.4)
Ostium primum ASD	29 (0.4)
Other abnormalities of atrioventricular and ventriculoarterial connection (ie, crisscross heart, isomerism/heterotaxy)	28 (0.3)
Anomalous coronary artery arising from the pulmonary artery	22 (0.3)
Infundibular right ventricular outflow obstruction	18 (0.2)
Peripheral pulmonary stenosis	16 (0.2)
Sinus venosus defect	16 (0.2)
Interrupted aortic arch	15 (0.2)
Sinus of Valsalva fistula/aneurysm	9 (0.1)
Anomalous aortic origin of a coronary artery from the opposite sinus	9 (0.1)
Aorto-left ventricular fistula	4 (0.05)
Mitral atresia	4 (0.05)
Straddling atrioventricular valve	2 (0.025)
Other defect of great complexity	57 (0.7)
Other defect of moderate complexity	157 (1.9)
Other defect of simple complexity	124 (1.5)
Cardiac surgery (n=8345)	6196 (74.2)
Catheter interventions (n=8381)	2820 (33.6)

ACHD AP, ACHD Anatomical and Physiological Classification; ASD, atrial septal defect; AVSD, atrio-ventricular septal defect; CCTGA, congenitally corrected transposition of the great arteries; d-TGA, dextro transposition of the great arteries; HCM, hypertrophic cardiomyopathy; TGA, transposition of the great arteries; VSD, ventricular septal defect.

### PROs in adults with CHD from 32 countries

**Figure 1:**
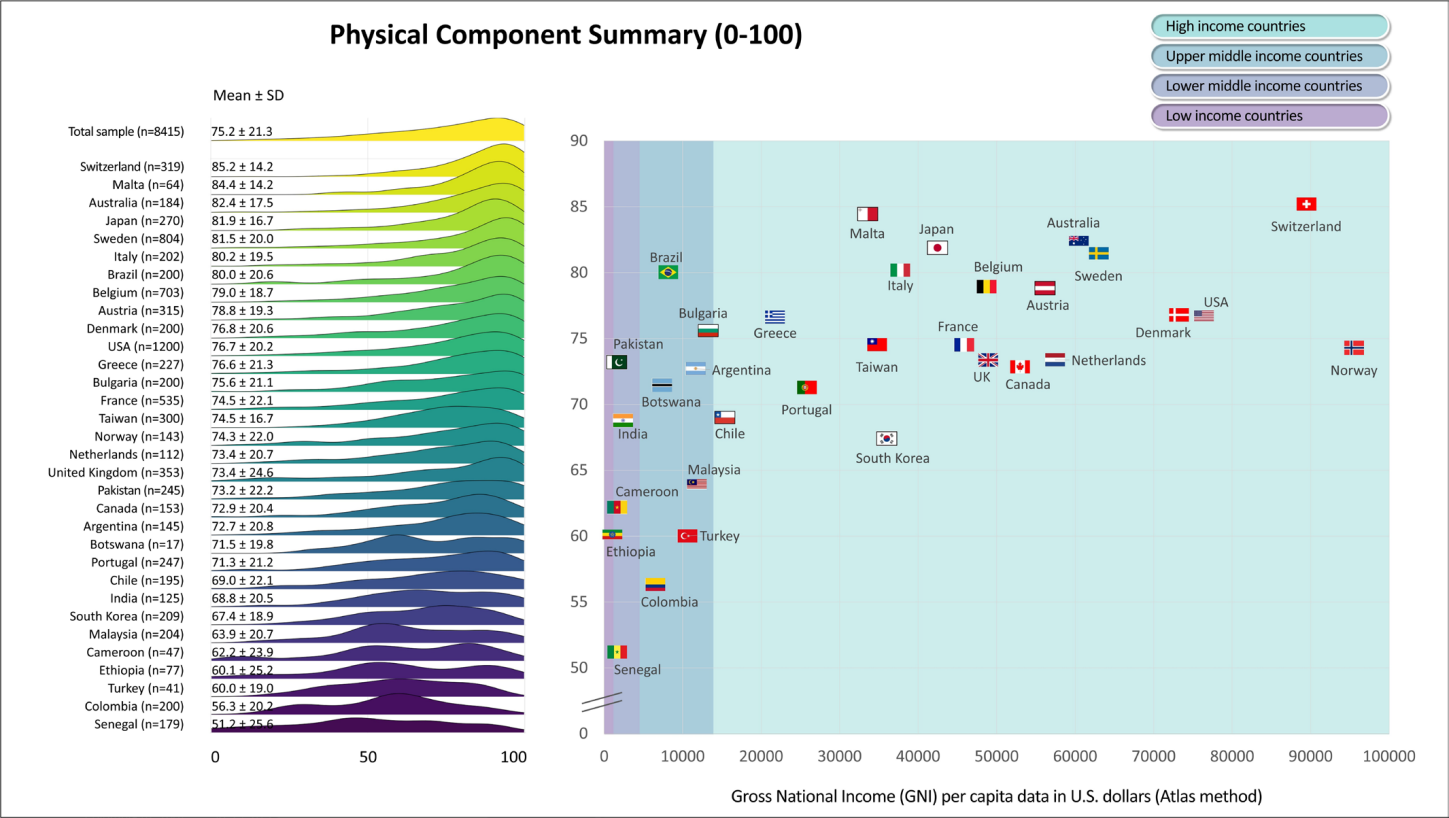
Physical functioning in adults with congenital heart disease from 32 countries.

**Figure 2:**
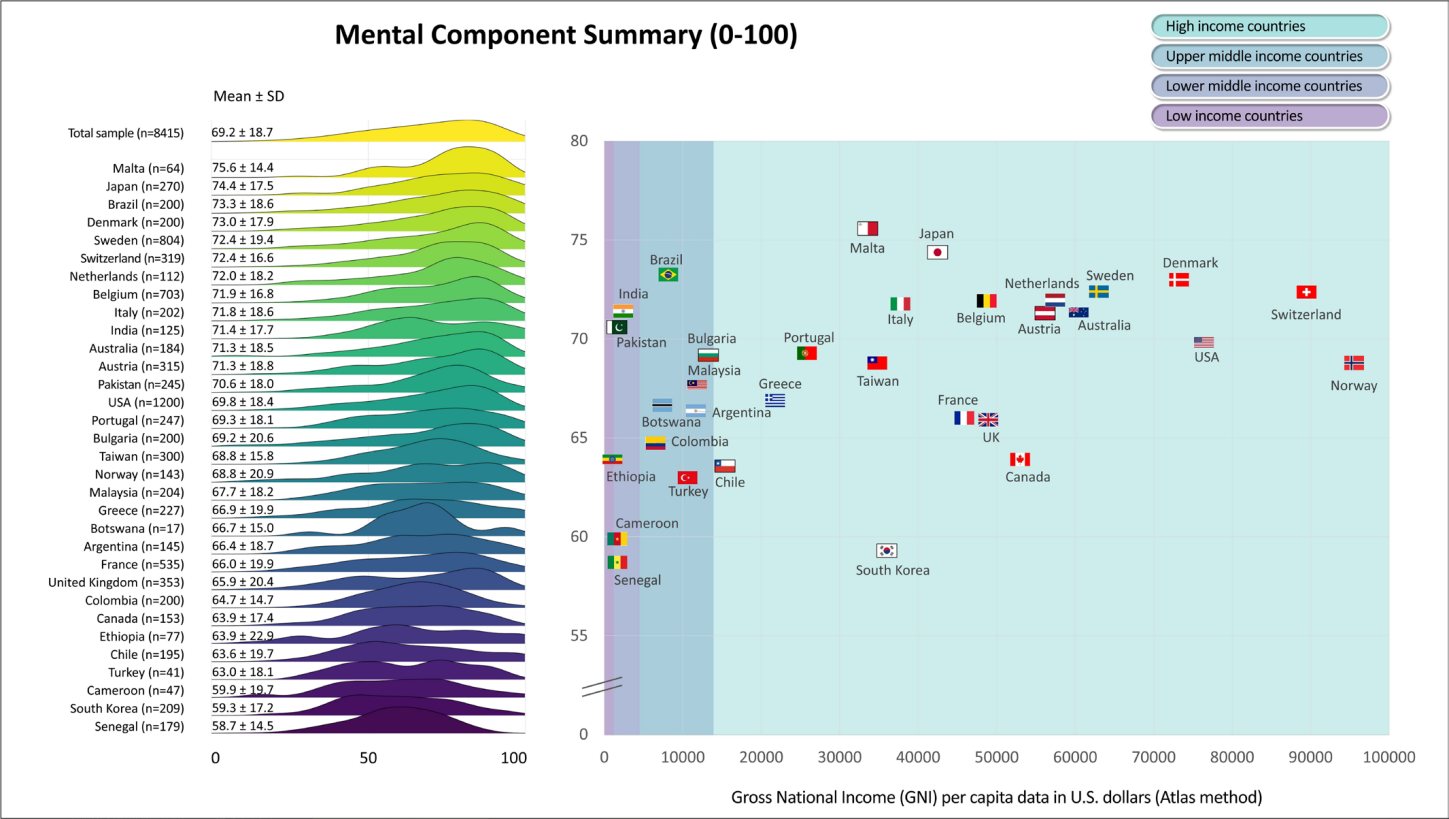
Mental health in adults with congenital heart disease from 32 countries.

**Figure 3:**
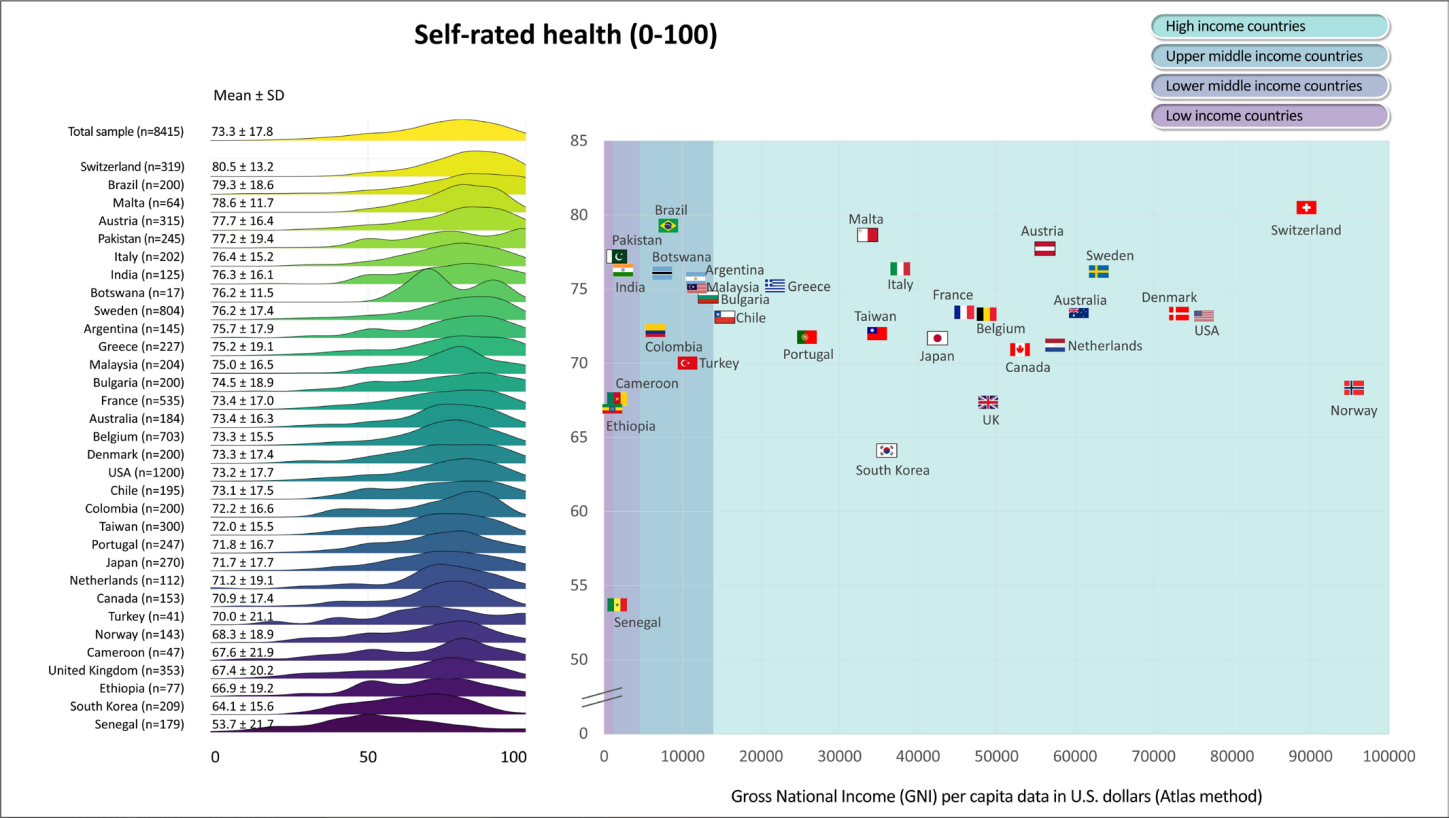
Self-rated health in adults with congenital heart disease from 32 countries.

**Figure 4:**
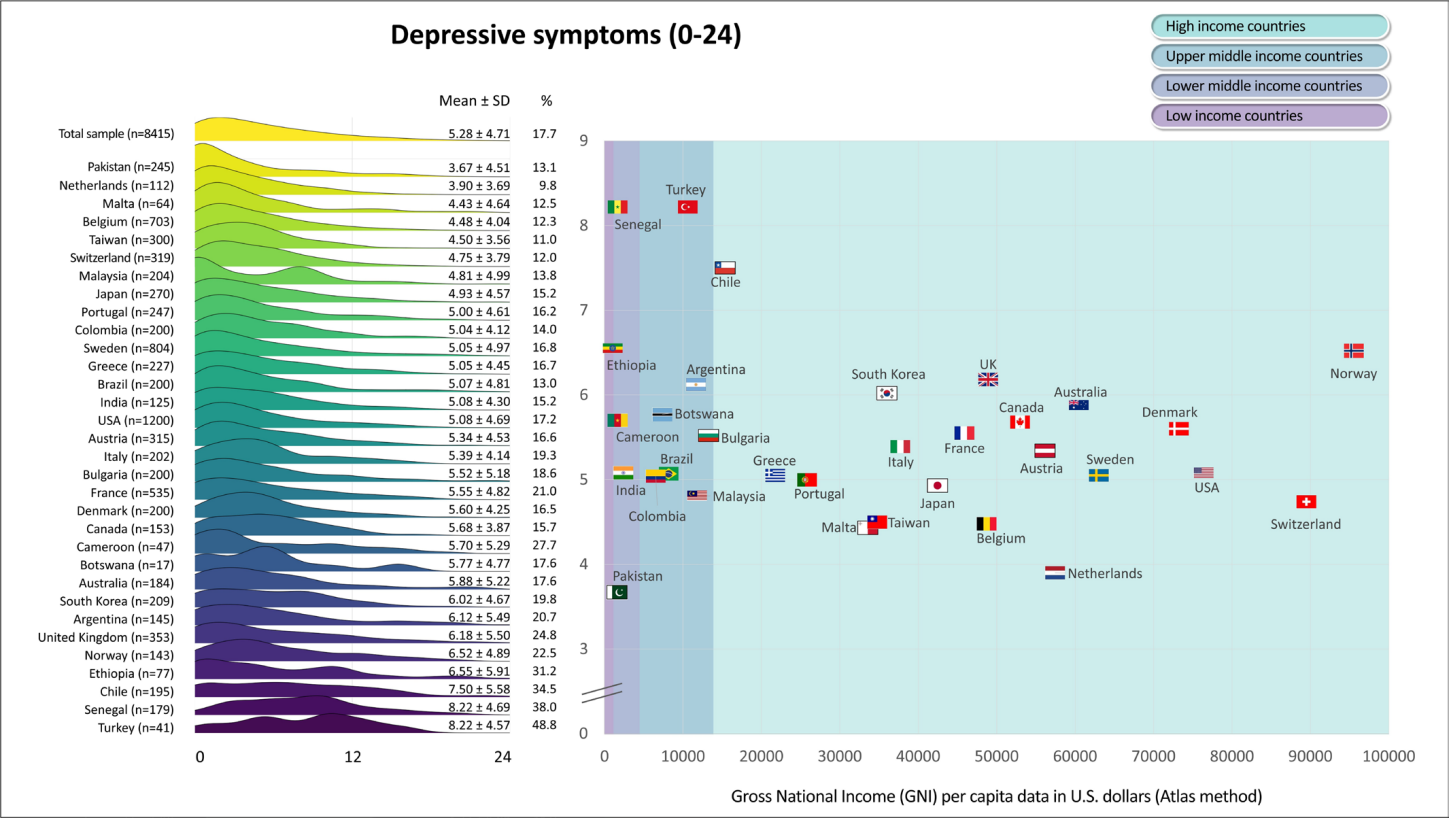
Depressive symptoms in adults with congenital heart disease from 32 countries.

**Figure 5:**
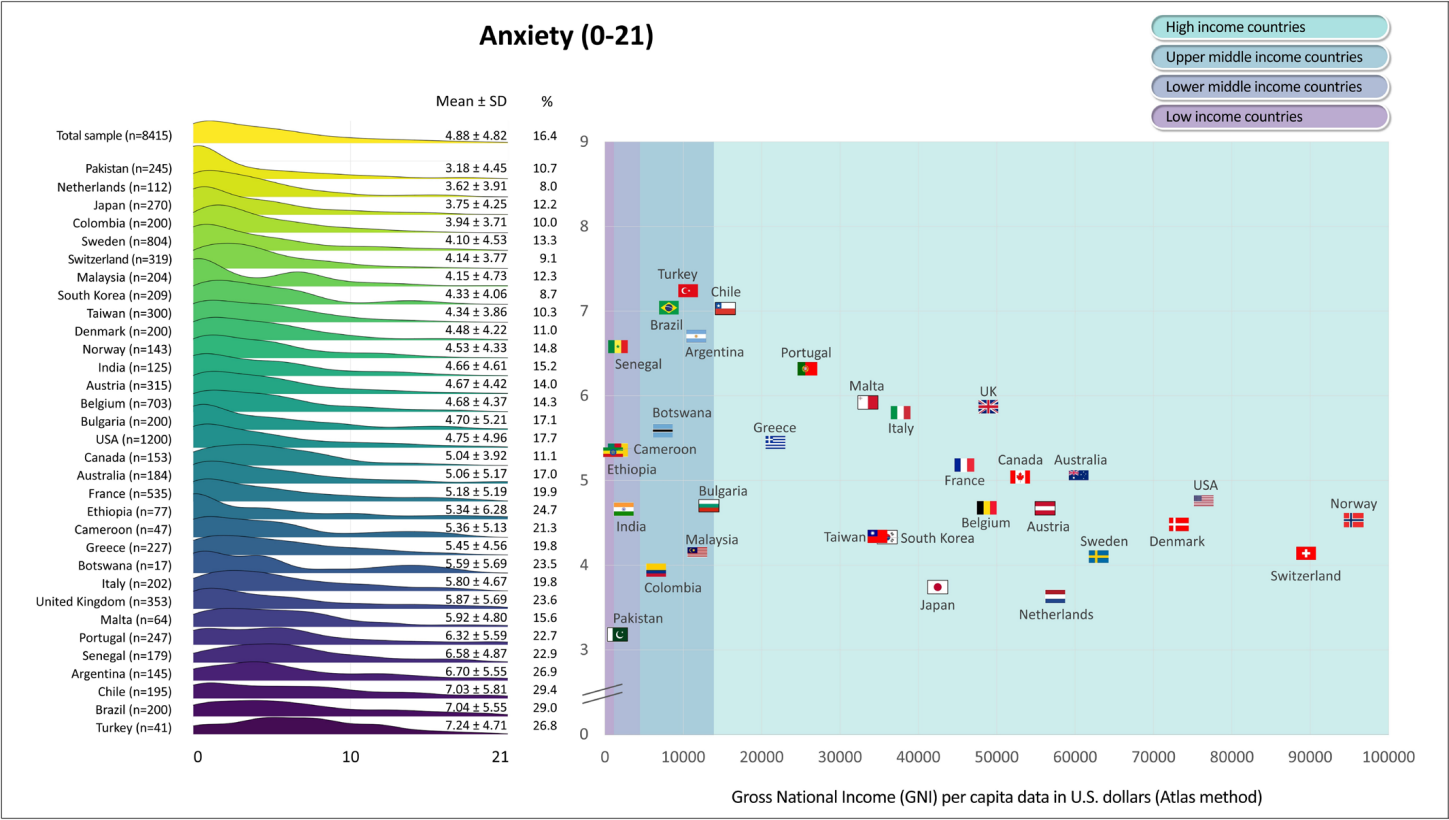
Anxiety in adults with congenital heart disease from 32 countries.

**Figure 6:**
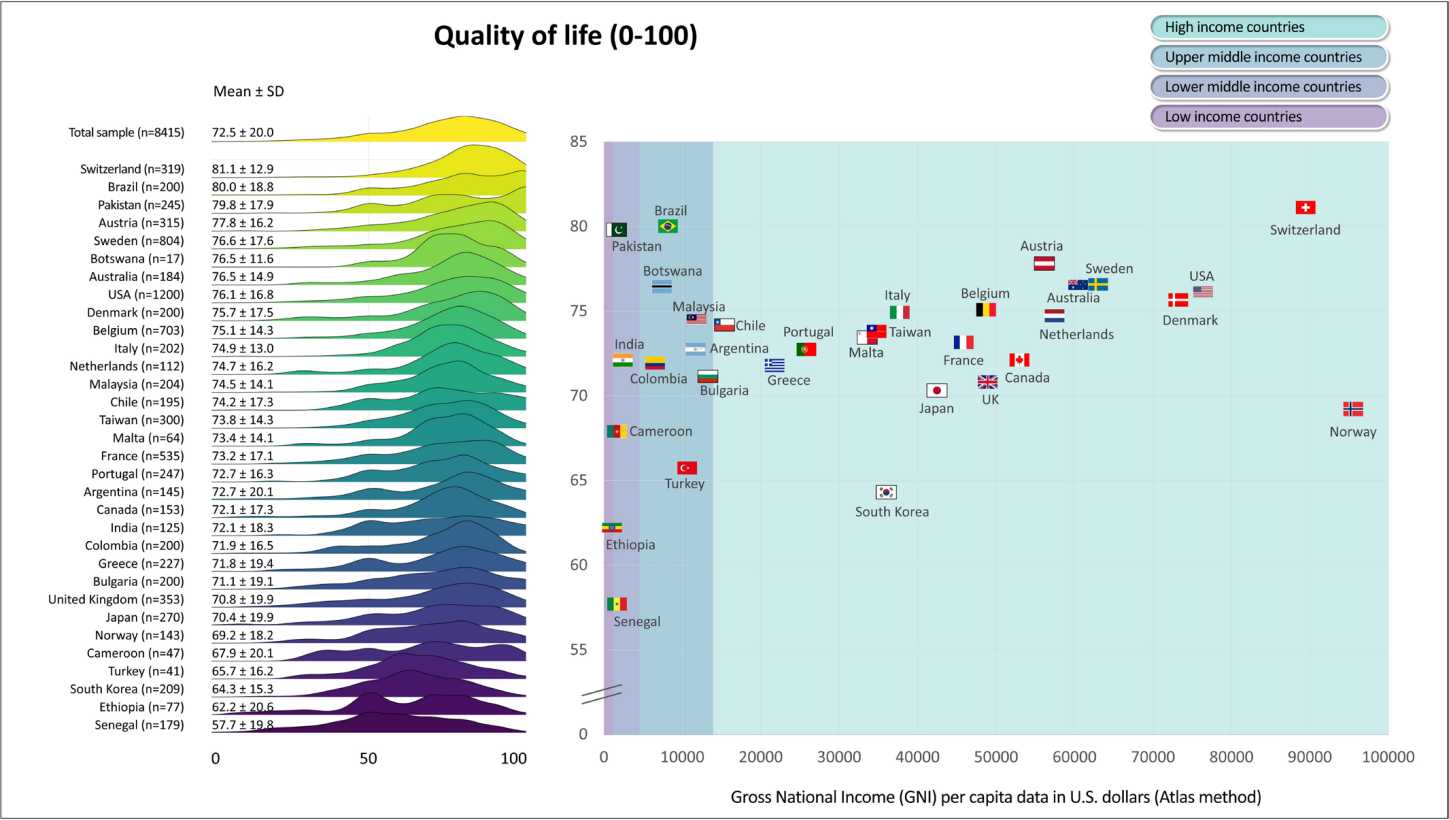
Quality of life health in adults with congenital heart disease from 32 countries.

Figures1^6^ display the mean scores (±SD) for the PROs for the total sample and for each participating country. The ridgeline plots in these figures visually depict density of the distribution. Notably, figures4  5 also provide the prevalence rates of depressive symptoms and moderate to severe anxiety symptoms. These results indicate large differences (with Cohen’s d effect size >0.8) in PROs between countries with the highest versus lowest scores (figures1^6^). Switzerland demonstrated the highest mean scores for physical functioning (figure 1), self-rated health (figure 3) and QoL (figure 6), while Senegal had the lowest scores. Conversely, patients from Malta demonstrated the highest mean scores for mental health (figure 2), with Senegal having the lowest scores. With regard to depressive symptoms and anxiety, Pakistan had the lowest mean scores, while Turkey had the highest scores (figures4  5). The prevalence of depressive symptoms in the overall sample was 17.7%, with a range from 9.8% in the Netherlands to 48.8% in Turkey (figure 4). Anxiety was observed in 16.4% of the sample, varying from 8.0% in the Netherlands to 29.4% in Chile (figure 5). Figures1^6^ show that the variability across middle and lower-income countries was higher for physical functioning, depressive symptoms, anxiety and QoL than high-income countries.

Utilising general linear mixed models adjusted for demographic (age; sex; employment status; marital status) and clinical (complexity of heart defect; physiological stage; cardiac surgery; catheter interventions) characteristics, a negative association was found between GNI and physical functioning (estimate=3.0; SE=0.4; p<0.001; figure 1), mental health (estimate=0.6; SE=0.3; p=0.042; figure 2) as well as QoL (estimate=0.8; SE=0.3; p=0.016; figure 6). The lower income countries tended to exhibit lower scores in these domains. However, no significant relationship was observed between GNI and other PROs (figures3^5^). Details of these analyses are provided in online supplemental table S3.

## Discussion

The global prevalence of CHD is increasing, primarily attributed to advancements in survival rates and population expansion beyond high-income nations.^19^ Consequently, healthcare systems will be confronted with an escalating number of adults requiring CHD care.^19^ From a global health perspective, understanding disparities in CHD outcomes holds importance, encompassing not only mortality but also other clinically relevant endpoints pertinent to patient well-being. APPROACH-IS II has simultaneously investigated PROs among individuals with CHD across diverse resource settings on all continents, employing a robust and standardised methodology.^9^ Such a comprehensive assessment is unprecedented within cardiac populations.

The present study revealed discernible disparities in physical functioning, mental health and QoL between patients in high-income countries compared with patients living in lower income countries. This confirms that inequalities in welfare are associated with disparities in PROs. Although patients from high-income countries were generally older, they exhibited superior physical health, mental health and QoL when compared with patients from low and middle-income countries. This is in line with the findings of the first APPROACH-IS project, in which a relationship between physical functioning and the Human Development Index was substantiated.^8^ This congruence is not surprising given that income level constitutes a facet of the Human Development Index, alongside metrics such as life expectancy and educational accessibility.^20^ The disparity in PROs across income classes may be due to inequalities in access to adequate CHD care as well as differences in healthcare priorities. Previous findings suggest that over 90% of CHD-affected individuals in low and middle-income countries receive no or suboptimal treatment,^21^ consequently exerting a deleterious influence on their health and overall well-being. Cultural attitudes toward health and the socioeconomic status of individual patients could also offer plausible rationales for the divergences in PROs across different income strata.^22^ Indeed, a nation’s income level may serve as an indirect proxy for gauging the influence of social determinants of health within populations and patient cohorts.

An interesting observation that emerged is the greater PRO variability among middle and low-income countries compared with their high-income counterparts, particularly in domains encompassing physical functioning, depressive symptoms, anxiety and QoL. In line with this, certain low-resource countries exhibited a relatively more favourable profile on mental health indicators, indicated by lower scores in depressive symptoms and anxiety. An illustrative instance is the cohort from Pakistan, which demonstrated the most favourable outcomes in terms of depressive symptoms and anxiety levels. These findings deviated from anticipated trends, contrasting with insights gleaned from a systematic review and meta-regression analysis, which identified Pakistan as harbouring the highest burden of mental health disorders in the South-East Asian region.^23^ Post hoc sensitivity analyses (data on file) by excluding Pakistan did not yield changes in our conclusions. The incongruity between the current study and prior research warrants further investigation.

### Methodological considerations

APPROACH-IS II is a unique project that investigated via a uniform methodology over 8000 patients from 32 high, middle and low income countries. We used valid and reliable instruments that were available in many languages to comprehensively assess PROs in an international setting. With these instruments, APPROACH-IS II covered most of the outcomes that are included in the standard set of PROs for CHD as recommended by the ICHOM.^24^ Although the project has been conducted mainly in the peri-pandemic phase of the COVID-19 pandemic, we were able to demonstrate the absence of pandemic-induced biases in the obtained scores.^11^ Indeed, we longitudinally compared prepandemic assessments (carried out between August 2019 and February 2020) with peri-pandemic evaluations (conducted from September 2020 to April 2021) in 716 adults with CHD from Belgium, Norway and South Korea. The analysis revealed minimal differences in PROs between the two periods, with effect sizes below the threshold of clinical significance (Cohen’s d <0.20). Additionally, no significant difference-in-differences was observed in PROs when comparing patients who had contracted COVID-19 with those who had not.^11^

Nonetheless, certain methodological constraints need to be acknowledged. First, the project had a cross-sectional research design, inherently limiting our ability to establish causality or ascertain the directionality of observed effects. Second, only patients who were physically and cognitively capable of completing surveys were included. This limitation precluded patients with significant neurodevelopmental deficits. Third, while we endeavoured to encompass a diverse representation of high, middle and low-income countries, an over-representation of high-income countries was inevitable, with merely one low-income country, Ethiopia, able to partake. Fourth, this project did not collect data in a control group. Therefore, we cannot infer how the data of patients with CHD differ from the general population. Fifth, for most participating countries, data from only one centre were available. It is unlikely that they are representative for the entire country. However, some countries (eg, Malta, Norway, Botswana) only have one specialised ACHD centre. Sixth, different data collection methods have been used (on-site, online, telephone interview). Posthoc analysis indicated that there were no significant differences in PRO scores across the different data collection methods. Seventh, akin to much PRO research, our project remains susceptible to survivorship bias, particularly accentuated within lower-income countries. In these regions, over 90% of CHD patients receive either no treatment or suboptimal care.^21^ Eighth, when cross-country comparisons are contemplated, ecological bias must be recognised, as national income levels may not invariably reflect individual well-being. In low-income countries like Ethiopia, a substantial prevalence of affluent patients is unlikely, while in high-income countries like, for instance, the USA, it is plausible that impoverished patients may be included, thereby deviating from the general economic profile of the country.

## Conclusion

APPROACH-IS II is the first international study that comprehensively assessed PROs in individuals with CHD from high-income, upper middle-income, lower middle-income and low-income countries. This study has revealed that patients from higher-income nations exhibited superior physical functioning, mental health and overall QoL. Large-scale international research endeavours, such as APPROACH-IS II, serve to illuminate the global burden of CHD for patients worldwide. Consequently, this research augments our understanding of the global burden of disease, a perspective predominantly characterised by assessments of mortality and morbidity.

## Supplementary material

10.1136/heartjnl-2024-325296 online supplemental file 1

## Data Availability

Data are available upon reasonable request.
