# Supplementary material for: Perceived health, psychological distress and quality of life in 8415 adults with congenital heart disease from 32 countries
Source: Heart. 2025 Jun 27;111(17):e325296. doi: 10.1136/heartjnl-2024-325296 (PMC12421120; doi:10.1136/heartjnl-2024-325296)
Supplement: online supplemental file 1 [file heartjnl-111-17-s001.docx]

SUPPLEMENTARY MATERIAL

**Table S1:** Demographic and clinical background variables for adults with congenital heart disease in different countries

| **Variables** | **Argentina** | **Australia** | **Austria** | **Belgium** | **Botswana** | **Brazil** | **Bulgaria** | **Cameroon** |
| --- | --- | --- | --- | --- | --- | --- | --- | --- |
| Sex |  |  |  |  |  |  |  |  |
| Men | 66 (45.5) | 86 (47.3) | 160 (51.0) | 368 (52.6) | 3 (17.6) | 76 (38.0) | 91 (45.5) | 18 (38.3) |
| Women | 79 (54.5) | 96 (52.7) | 154 (49.0) | 332 (47.4) | 14 (82.4) | 124 (62.0) | 108 (54.0) | 29 (61.7) |
| Other |  |  |  |  |  |  | 1 (0.5) |  |
| Median age in years (IQR) | 34 (25.25-43.75) | 28 (22-35) | 35 (28-45) | 28 (26-38) | 34 (23.5-41) | 30 (23-47) | 24 (21-32) | 31 (20-34) |
| Background |  |  |  |  |  |  |  |  |
| White or Caucasian | 58 (40.0) | 141 (76.6) | 299 (94.9) | 681 (96.9) |  | 173 (86.5) | 186 (93.0) |  |
| Asian |  | 16 (8.7) | 4 (1.3) | 9 (1.3) |  |  |  |  |
| Hispanic or Latino | 84 (57.9) | 1 (0.5) | 1 (0.3) |  |  | 2 (1.0) | 1 (0.5) |  |
| Black or African-American |  | 1 (0.5) | 1 (0.3) |  | 17 (100.0) | 25 (12.5) |  | 36 (76.6) |
| Middle-Eastern or Arabic |  | 4 (2.2) | 4 (1.3) | 4 (0.6) |  |  |  |  |
| Other | 3 (2.1) | 21 (11.4) | 6 (1.9) | 9 (1.3) |  |  | 13 (6.5) | 11 (23.4) |
| Educational level |  |  |  |  |  |  |  |  |
| Less than high school | 33 (22.8) | 38 (21.0) | 157 (51.1) | 39 (5.6) | 8 (47.1) | 48 (24.1) | 14 (7.0) | 12 (25.5) |
| High school | 47 (32.4) | 83 (45.9) | 53 (17.3) | 299 (43.1) | 8 (47.1) | 125 (62.5) | 114 (57.0) | 12 (25.5) |
| Bachelor or College degree | 35 (24.1) | 56 (30.9) | 42 (13.7) | 223 (32.2) | 1 (5.9) | 26 (13.0) | 32 (16.0) | 16 (34.0) |
| Master degree or higher | 30 (20.7) | 4 (2.2) | 55 (17.9) | 132 (19.0) |  |  | 40 (20.0) | 7 (14.9) |
| Employment status |  |  |  |  |  |  |  |  |
| Part-time or full-time work | 68 (47.2) | 123 (67.2) | 211 (67.4) | 497 (71.4) | 5 (29.4) | 105 (52.5) | 97 (48.5) | 18 (38.3) |
| Job seeking, unemployed, or disability | 28 (19.4) | 20 (10.9) | 28 (8.9) | 55 (7.9) | 9 (52.9) | 35 (17.5) | 32 (16.0) | 7 (14.9) |
| Homemaker or retired | 24 (16.7) | 3 (1.6) | 28 (8.9) | 50 (7.2) |  | 53 (26.5) | 8 (4.0) | 1 (2.1) |
| Full-time student | 15 (10.4) | 33 (18.0) | 13 (4.2) | 40 (5.7) | 3 (17.6) | 6 (3.0) | 38 (19.0) | 8 (17.0) |
| Other | 9 (6.3) | 4 (2.2) | 33 (10.5) | 54 (7.8) |  | 1 (0.5) | 25 (12.5) |  |
| Marital status |  |  |  |  |  |  |  |  |
| Married or living with partner | 64 (44.1) | 74 (40.4) | 183 (58.5) | 359 (51.7) | 5 (29.4) | 86 (43.0) | 68 (34.0) | 16 (34.0) |
| Never married | 74 (51.0) | 103 (56.3) | 115 (36.7) | 307 (44.2) | 12 (70.6) | 89 (44.5) | 125 (62.5) | 27 (57.4) |
| Divorced or widowed | 7 (4.8) | 5 (2.7) | 14 (4.5) | 27 (3.9) |  | 25 (12.5) | 7 (3.5) | 2 (4.3) |
| Other |  | 1 (0.5) | 1 (0.3) | 1 (0.1) |  |  |  | 2 (4.3) |
| Anatomical complexity of ACHD AP |  |  |  |  |  |  |  |  |
| Simple | 12 (12.4) | 31 (16.8) | 8 (2.5) | 112 (16.0) | 6 (37.5) | 66 (33.0) | 18 (9.0) | 25 (54.3) |
| Moderate | 70 (72.2) | 113 (61.4) | 211 (67.0) | 439 (62.7) | 10 (62.5) | 104 (52.0) | 127 (63.8) | 19 (41.3) |
| Complex | 15 (15.5) | 40 (21.7) | 96 (30.5) | 149 (21.3) |  | 30 (15.0) | 54 (27.1) | 2 (4.3) |
| Physiological stage of ACHD AP |  |  |  |  |  |  |  |  |
| Stage A | 9 (9.3) | 21 (11.4) | 26 (8.7) | 85 (12.2) | 4 (26.7) | 31 (15.7) | 13 (6.6) | 10 (22.7) |
| Stage B | 27 (27.8) | 84 (45.7) | 96 (32.0) | 228 (32.8) | 8 (53.3) | 91 (46.2) | 110 (56.1) | 13 (29.5) |
| Stage C | 55 (56.7) | 76 (41.3) | 153 (51.0) | 340 (48.9) | 3 (20.0) | 62 (31.5) | 49 (25.0) | 19 (43.2) |
| Stage D | 6 (6.2) | 3 (1.6) | 25 (8.3) | 42 (6.0) |  | 13 (6.6) | 24 (12.2) | 2 (4.5) |
| New York Heart Association assessment |  |  |  |  |  |  |  |  |
| Class I | 54 (55.7) | 156 (84.8) | 190 (60.5) | 597 (85.9) | 7 (43.8) | 157 (78.5) | 58 (29.1) | 24 (53.3) |
| Class II | 35 (36.1) | 25 (13.6) | 94 (29.9) | 86 (12.4) | 8 (50.0) | 34 (17.0) | 110 (55.3) | 18 (40.0) |
| Class III | 8 (8.2) | 3 (1.6) | 25 (8.0) | 12 (1.7) | 1 (6.3) | 9 (4.5) | 30 (15.1) | 3 (6.7) |
| Class IV |  |  | 5 (1.6) |  |  |  | 1 (0.5) |  |
| Cardiac surgery | 65 (45.8) | 128 (69.9) | 239 (76.4) | 494 (70.3) | 6 (35.3) | 98 (49.0) | 162 (81.0) | 28 (59.6) |
| Catheter interventions | 38 (26.4) | 62 (33.7) | 33 (10.5) | 252 (35.8) | 0 (0.0) | 36 (18.0) | 53 (26.6) | 16 (34.0) |

**Table S1:** Demographic and clinical background variables for adults with congenital heart disease in different countries (cont.)

| **Variables** | **Canada** | **Chile** | **Colombia** | **Denmark** | **Ethiopia** | **France** | **Greece** | **India** |
| --- | --- | --- | --- | --- | --- | --- | --- | --- |
| Sex |  |  |  |  |  |  |  |  |
| Men | 80 (52.3) | 91 (46.9) | 81 (40.5) | 95 (47.5) | 24 (31.2) | 251 (46.9) | 123 (54.2) | 61 (48.8) |
| Women | 73 (47.7) | 103 (53.1) | 119 (59.5) | 105 (52.5) | 53 (68.8) | 282 (52.7) | 104 (45.8) | 64 (51.2) |
| Other |  |  |  |  |  | 2 (0.4) |  |  |
| Median age in years (IQR) | 42 (34-53.75) | 27 (23-36) | 25 (22-30) | 36 (25-47.75) | 28 (22-35) | 33 (26-42) | 28 (23-39) | 23 (20-28) |
| Background |  |  |  |  |  |  |  |  |
| White or Caucasian | 135 (88.2) | 2 (1.0) |  | 181 (91.0) |  | 444 (84.1) | 195 (85.9) |  |
| Asian | 6 (3.9) |  |  | 4 (2.0) |  | 7 (1.3) |  | 125 (100.0) |
| Hispanic or Latino | 1 (0.7) | 186 (95.4) | 196 (98.0) | 1 (0.5) |  | 4 (0.8) |  |  |
| Black or African-American | 1 (0.7) | 1 (0.5) | 4 (2.0) | 1 (0.5) | 76 (98.7) | 24 (4.5) |  |  |
| Middle Eastern or Arabic | 3 (2.0) |  |  | 7 (3.5) |  | 44 (8.3) |  |  |
| Other | 7 (4.6) | 6 (3.1) |  | 5 (2.5) | 1 (1.3) | 5 (0.9) | 32 (14.1) |  |
| Educational level |  |  |  |  |  |  |  |  |
| Less than high school | 14 (9.3) | 35 (18.7) | 90 (45.9) | 41 (20.8) | 44 (61.1) | 87 (16.9) | 28 (12.3) | 5 (4.0) |
| High school | 57 (37.7) | 109 (58.3) | 81 (41.3) | 82 (41.6) | 17 (23.6) | 220 (42.8) | 106 (46.7) | 51 (40.8) |
| Bachelor or College degree | 73 (48.3) | 39 (20.9) | 20 (10.2) | 42 (21.3) | 9 (12.5) | 116 (22.6) | 74 (32.6) | 54 (43.2) |
| Master’s degree or higher | 7 (4.6) | 4 (2.1) | 5 (2.6) | 32 (16.0) | 2 (2.8) | 91 (17.7) | 19 (8.4) | 15 (12.0) |
| Employment status |  |  |  |  |  |  |  |  |
| Part-time or full-time work | 107 (69.9) | 72 (37.7) | 60 (30.2) | 98 (49.2) | 25 (33.8) | 289 (54.7) | 85 (37.4) | 35 (28.0) |
| Job seeking, unemployed, or disability | 20 (13.1) | 51 (26.7) | 72 (36.2) | 27 (13.6) | 15 (20.3) | 105 (19.9) | 56 (24.7) | 34 (27.2) |
| Homemaker or retired | 18 (11.8) | 20 (10.5) | 36 (18.1) | 11 (5.5) | 13 (17.6) | 47 (8.9) | 14 (6.2) | 2 (1.6) |
| Full-time student | 2 (1.3) | 37 (19.4) | 13 (6.5) | 33 (16.6) | 15 (20.3) | 41 (7.8) | 50 (22.0) | 53 (42.4) |
| Other | 6 (3.9) | 11 (5.8) | 18 (9.0) | 30 (15.1) | 6 (8.1) | 46 (8.7) | 22 (9.7) | 1 (0.8) |
| Marital status |  |  |  |  |  |  |  |  |
| Married or living with partner | 98 (64.1) | 52 (26.9) | 80 (40.8) | 109 (54.5) | 26 (33.8) | 250 (47.1) | 86 (37.9) | 21 (16.8) |
| Never married | 42 (27.5) | 131 (67.9) | 106 (54.1) | 78 (39.0) | 48 (62.3) | 257 (48.4) | 135 (59.5) | 104 (83.2) |
| Divorced or widowed | 13 (8.5) | 10 (5.2) | 10 (5.1) | 11 (5.5) | 3 (3.9) | 22 (4.1) | 4 (1.8) |  |
| Other |  |  |  | 2 (1.0) |  | 2 (0.4) | 2 (0.9) |  |
| Anatomical complexity of ACHD AP |  |  |  |  |  |  |  |  |
| Simple | 12 (7.8) | 32 (16.4) | 46 (23.0) | 8 (4.0) | 14 (35.0) | 35 (6.6) | 33 (14.5) | 28 (22.4) |
| Moderate | 101 (66.0) | 93 (47.7) | 114 (57.0) | 126 (63.0) | 23 (57.5) | 307 (57.8) | 119 (52.4) | 47 (37.6) |
| Complex | 40 (26.1) | 70 (35.9) | 40 (20.0) | 66 (33.0) | 3 (7.5) | 189 (35.6) | 75 (33.0) | 50 (40.0) |
| Physiological stage of ACHD AP |  |  |  |  |  |  |  |  |
| Stage A | 25 (16.7) | 12 (6.2) | 8 (4.0) | 18 (9.0) | 3 (7.9) | 49 (9.3) | 9 (4.0) | 38 (30.4) |
| Stage B | 25 (16.7) | 71 (36.4) | 51 (25.5) | 64 (32.2) | 11 (28.9) | 117 (22.2) | 70 (30.8) | 24 (19.2) |
| Stage C | 96 (64.0) | 98 (50.3) | 96 (48.0) | 98 (49.2) | 15 (39.5) | 311 (59.1) | 137 (60.4) | 45 (36.0) |
| Stage D | 4 (2.7) | 14 (7.2) | 45 (22.5) | 19 (9.5) | 9 (23.7) | 49 (9.3) | 11 (4.8) | 18 (14.4) |
| New York Heart Association assessment |  |  |  |  |  |  |  |  |
| Class I | 79 (65.3) | 155 (79.5) | 97 (48.7) | 136 (68.7) | 20 (50.0) | 340 (63.6) | 169 (76.1) | 76 (60.8) |
| Class II | 32 (26.4) | 25 (12.8) | 72 (36.2) | 56 (28.3) | 17 (42.5) | 159 (29.7) | 45 (20.3) | 41 (32.8) |
| Class III | 10 (8.3) | 15 (7.7) | 29 (14.6) | 6 (3.0) | 3 (7.5) | 32 (6.0) | 8 (3.6) | 7 (5.6) |
| Class IV |  |  | 1 (0.) |  |  | 4 (0.7) |  | 1 (0.8) |
| Cardiac surgery | 125 (85.0) | 162 (83.5) | 104 (53.6) | 168 (84.4) | 1 (1.3) | 425 (79.6) | 156 (69.6) | 88 (71.0) |
| Catheter interventions | 24 (15.9) | 68 (34.9) | 120 (60.9) | 59 (29.6) | 3 (3.9) | 192 (36.0) | 52 (22.9) | 40 (32.3) |

**Table S1:** Demographic and clinical background variables for adults with congenital heart disease in different countries (cont.)

| **Variables** | **Italy** | **Japan** | **Malaysia** | **Malta** | **Netherlands** | **Norway** | **Pakistan** | **Portugal** |
| --- | --- | --- | --- | --- | --- | --- | --- | --- |
| Sex |  |  |  |  |  |  |  |  |
| Men | 111 (55.0) | 128 (47.4) | 70 (34.5) | 32 (50.0) | 51 (45.5) | 56 (39.4) | 123 (50.4) | 108 (43.7) |
| Women | 91 (45.0) | 142 (52.6) | 133 (65.5) | 32 (50.0) | 61 (54.5) | 86 (60.6) | 121 (49.6) | 139 (56.3) |
| Other |  |  |  |  |  |  |  |  |
| Median age in years (IQR) | 28 (21-47) | 37 (27-48.25) | 28 (24-36.75) | 28.5 (24-38) | 40.5 (32-51) | 38 (29-51) | 29 (23-37.5) | 36 (27-46) |
| Background |  |  |  |  |  |  |  |  |
| White or Caucasian | 194 (96.0) | 25 (9.3) |  | 64 (100.0) | 103 (92.0) | 136 (95.1) |  | 244 (98.8) |
| Asian | 2 (1.0) | 243 (90.0) | 204 (100.0) |  | 1 (0.9) | 3 (2.1) | 245 (100.0) | 1 (0.4) |
| Hispanic or Latino | 5 (2.5) |  |  |  |  | 1 (0.7) |  |  |
| Black or African-American |  |  |  |  |  | 1 (0.7) |  | 2 (0.8) |
| Middle Eastern or Arabic |  |  |  |  |  |  |  |  |
| Other | 1 (0.5) | 2 (0.7) |  |  | 8 (7.1) | 2 (1.4) |  |  |
| Educational level |  |  |  |  |  |  |  |  |
| Less than high school | 26 (12.9) | 17 (6.3) | 4 (2.0) | 11 (17.5) | 8 (7.1) | 10 (7.0) | 72 (29.9) | 83 (33.9) |
| High school | 127 (63.5) | 112 (41.6) | 94 (46.1) | 32 (50.8) | 59 (52.7) | 60 (42.0) | 69 (28.6) | 97 (39.6) |
| Bachelor or College degree | 33 (16.5) | 133 (49.4) | 58 (28.4) | 14 (22.2) | 31 (27.7) | 33 (23.1) | 62 (25.7) | 42 (17.1) |
| Master’s degree or higher | 14 (7.0) | 7 (2.6) | 48 (23.5) | 6 (9.5) | 14 (12.5) | 40 (28.0) | 38 (15.8) | 23 (9.4) |
| Employment status |  |  |  |  |  |  |  |  |
| Part-time or full-time work | 107 (53.2) | 177 (65.6) | 103 (50.5) | 51 (79.7) | 65 (58.0) | 74 (51.7) | 91 (37.1) | 148 (60.9) |
| Job seeking, unemployed, or disability | 26 (12.9) | 27 (10.0) | 32 (15.7) | 1 (1.6) | 15 (13.4) | 44 (30.8) | 44 (18.0) | 49 (20.2) |
| Homemaker or retired | 14 (7.0) | 32 (11.9) | 39 (19.1) | 2 (3.1) | 11 (9.8) | 8 (5.6) | 58 (23.7) | 19 (7.8) |
| Full-time student | 25 (12.4) | 10 (3.7) | 7 (3.4) | 9 (14.1) | 7 (6.3) | 6 (4.2) | 44 (18.0) | 12 (4.9) |
| Other | 29 (14.4) | 24 (8.9) | 23 (11.3) | 1 (1.6) | 14 (12.5) | 11 (7.7) |  | 15 (6.2) |
| Marital status |  |  |  |  |  |  |  |  |
| Married or living with partner | 68 (34.0) | 136 (50.4) | 97 (47.8) | 25 (41.0) | 77 (68.8) | 88 (62.0) | 133 (54.3) | 116 (47.0) |
| Never married | 126 (63.0) | 120 (44.4) | 100 (49.3) | 32 (52.5) | 30 (26.8) | 40 (28.2) | 109 (44.5) | 112 (45.3) |
| Divorced or widowed | 4 (2.0) | 13 (4.8) | 6 (3.0) | 4 (6.6) | 4 (3.6) | 14 (9.9) | 3 (1.2) | 19 (7.7) |
| Other | 2 (1.0) | 1 (0.4) |  |  | 1 (0.9) |  |  |  |
| Anatomical complexity of ACHD AP |  |  |  |  |  |  |  |  |
| Simple | 78 (38.6) | 83 (30.7) | 61 (30.0) | 5 (7.8) | 2 (1.8) | 8 (6.0) | 104 (45.0) | 15 (6.1) |
| Moderate | 103 (51.0) | 121 (44.8) | 119 (58.6) | 50 (78.1) | 70 (62.5) | 82 (61.7) | 105 (45.5) | 179 (72.5) |
| Complex | 21 (10.4) | 66 (24.4) | 23 (11.3) | 9 (14.1) | 40 (35.7) | 43 (32.3) | 22 (9.5) | 53 (21.5) |
| Physiological stage of ACHD AP |  |  |  |  |  |  |  |  |
| Stage A | 51 (25.8) | 46 (17.8) | 36 (17.7) | 13 (20.3) | 6 (5.4) | 1 (0.7) | 7 (3.6) | 12 (4.9) |
| Stage B | 74 (37.4) | 80 (31.0) | 55 (27.1) | 23 (35.9) | 27 (24.1) | 27 (19.9) | 103 (52.6) | 65 (26.6) |
| Stage C | 67 (33.8) | 114 (44.2) | 91 (44.8) | 27 (42.2) | 75 (67.0) | 104 (76.5) | 66 (33.7) | 162 (66.4) |
| Stage D | 6 (3.0) | 18 (7.0) | 21 (10.3) | 1 (1.6) | 4 (3.6) | 4 (2.9) | 20 (10.2) | 5 (2.0) |
| New York Heart Association assessment |  |  |  |  |  |  |  |  |
| Class I | 132 (65.3) | 226 (84.6) | 143 (70.1) | 62 (98.4) | 77 (70..0) | 77 (56.6) | 144 (69.9) | 198 (80.2) |
| Class II | 50 (24.8) | 38 (14.2) | 56 (27.5) | 1 (1.6) | 29 (26.4) | 46 (33.8) | 49 (23.8) | 47 (19.0) |
| Class III | 20 (9.9) | 3 (1.1) | 5 (2.5) |  | 3 (2.7) | 13 (9.6) | 11 (5.6) | 2 (0.8) |
| Class IV |  |  |  |  | 1 (0.9) |  | 2 (1.0) |  |
| Cardiac surgery | 112 (55.4) | 231 (85.6) | 117 (57.6) | 40 (62.5) | 100 (89.3) | 123 (87.2) | 141 (57.6) | 211 (85.4) |
| Catheter interventions | 83 (41.1) | 56 (20.7) | 53 (26.2) | 20 (31.3) | 22 (19.6) | 119 (83.8) | 78 (31.8) | 38 (15.4) |

**Table S1:** Demographic and clinical background variables for adults with congenital heart disease in different countries (cont.)

| **Variables** | **Senegal** | **South Korea** | **Sweden** | **Switzerland** | **Taiwan** | **Turkey** | **UK** | **USA** |
| --- | --- | --- | --- | --- | --- | --- | --- | --- |
| Sex |  |  |  |  |  |  |  |  |
| Men | 79 (44.1) | 103 (49.8) | 416 (51.8) | 145 (45.6) | 103 (34.3) | 23 (56.1) | 179 (50.9) | 463 (38.8) |
| Women | 100 (55.9) | 104 (50.2) | 387 (48.2) | 172 (54.1) | 197 (65.7) | 18 (43.9) | 172 (48.9) | 728 (61.0) |
| Other |  |  |  | 1 (0.3) |  |  | 1 (0.3) | 2 (0.2) |
| Median age in years (IQR) | 21 (19-28) | 30 (22-44) | 40 (30-53) | 32 (26-42) | 32 (24-38) | 24 (20-33.5) | 37 (30-47) | 35 (28-47) |
| Background |  |  |  |  |  |  |  |  |
| White or Caucasian |  |  | 752 (93.5) | 302 (94.7) | 2 (0.7) |  | 323 (92.0) | 938 (78.4) |
| Asian |  | 209 (100.0) | 9 (1.1) | 7 (2.2) | 298 (99.3) | 1 (2.4) | 11 (3.1) | 66 (5.5) |
| Hispanic or Latino |  |  | 3 (0.4) |  |  |  | 1 (0.3) | 79 (6.6) |
| Black or African-American | 179 (100.0) |  | 3 (0.4) | 4 (1.3) |  |  | 1 (0.3) | 74 (6.2) |
| Middle Eastern or Arabic |  |  | 10 (1.2) | 3 (0.9) |  | 1 (2.4) | 3 (0.9) | 9 (0.8) |
| Other |  |  | 27 (3.4) | 3 (0.9) |  | 39 (95.1) | 12 (3.4) | 31 (2.6) |
| Educational level |  |  |  |  |  |  |  |  |
| Less than high school | 131 (73.2) | 5 (2.4) | 79 (10.0) | 17 (5.4) | 6 (2.0) | 12 (29.3) | 38 (10.8) | 29 (2.4) |
| High school | 39 (21.8) | 92 (44.9) | 385 (48.9) | 164 (51.9) | 103 (34.3) | 18 (43.9) | 148 (42.0) | 429 (36.1) |
| Bachelor or College degree | 6 (3.4) | 65 (31.7) | 211 (26.8) | 72 (22.8) | 140 (46.7) | 9 (22.0) | 132 (37.5) | 454 (38.2) |
| Master’s degree or higher | 3 (1.7) | 43 (21.0) | 113 (14.3) | 63 (19.9) | 51 (17.0) | 2 (4.9) | 34 (9.7) | 278 (23.4) |
| Employment status |  |  |  |  |  |  |  |  |
| Part-time or full-time work | 40 (22.3) | 74 (36.1) | 545 (68.7) | 227 (71.4) | 200 (66.7) | 17 (41.5) | 247 (70.6) | 793 (66.3) |
| Job seeking, unemployed, or disability | 30 (16.9) | 50 (24.4) | 79 (10.0) | 21 (6.6) | 28 (9.3) | 11 (26.8) | 41 (11.7) | 175 (14.6) |
| Homemaker or retired | 59 (33.1) | 25 (12.2) | 72 (9.1) | 16 (5.0) | 21 (7.0) | 3 (7.3) | 29 (8.3) | 100 (8.4) |
| Full-time student | 22 (15.2) | 55 (26.8) | 27 (3.4) | 21 (6.6) | 42 (14.0) | 10 (24.4) | 16 (4.6) | 74 (6.2) |
| Other | 27 (15.2) | 1 (0.5) | 70 (8.8) | 33 (10.4) | 9 (3.0) |  | 17 (4.9) | 54 (4.5) |
| Marital status |  |  |  |  |  |  |  |  |
| Married or living with partner | 25 (14.0) | 68 (32.7) | 509 (63.8) | 158 (49.7) | 107 (35.7) | 13 (31.7) | 206 (58.5) | 668 (55.8) |
| Never married | 145 (81.0) | 131 (63.0) | 222 (27.8) | 152 (47.8) | 185 (61.7) | 27 (65.9) | 131 (37.2) | 446 (37.2) |
| Divorced or widowed | 9 (5.0) | 9 (4.3) | 61 (7.6) | 8 (2.5) | 7 (2.3) | 1 (2.4) | 14 (4.0) | 82 (6.8) |
| Other |  |  | 6 (0.8) |  | 1 (0.3) |  | 1 (0.3) | 2 (0.2) |
| Anatomical complexity of ACHD AP |  |  |  |  |  |  |  |  |
| Simple | 10 (18.9) | 10 (4.8) | 69 (8.7) | 22 (6.9) | 112 (37.6) | 7 (17.1) | 11 (3.3) | 107 (8.7) |
| Moderate | 38 (71.7) | 78 (37.5) | 507 (64.3) | 215 (67.8) | 134 (45.0) | 19 (46.3) | 218 (64.9) | 679 (56.7) |
| Complex | 5 (9.4) | 120 (57.7) | 213 (27.0) | 80 (25.2) | 52 (17.4) | 15 (36.6) | 107 (31.8) | 415 (34.6) |
| Physiological stage of ACHD AP |  |  |  |  |  |  |  |  |
| Stage A |  | 1 (0.5) | 120 (15.1) | 36 (11.3) | 31 (10.5) | 2 (4.9) | 21 (6.3) | 144 (12.4) |
| Stage B | 14 (26.4) | 26 (12.4) | 242 (30.4) | 77 (24.2) | 101 (34.1) | 2 (4.9) | 91 (27.1) | 278 (23.9) |
| Stage C | 29 (54.7) | 152 (72.5) | 382 (48.1) | 189 (59.4) | 155 (52.4) | 25 (61.0) | 192 (57.1) | 683 (58.8) |
| Stage D | 10 (18.9) | 30 (14.4) | 51 (6.4) | 16 (5.0) | 9 (3.0) | 13 (29.3) | 32 (9.5) | 56 (4.8) |
| New York Heart Association assessment |  |  |  |  |  |  |  |  |
| Class I | 1 (1.9) | 4 (1.9) | 627 (78.6) | 248 (77.7) | 192 (64.4) | 8 (20.0) | 223 (66.2) | 756 (63.9) |
| Class II | 25 (47.2) | 191 (91.4) | 144 (18.0) | 66 (20.7) | 88 (29.5) | 23 (57.5) | 85 (25.2) | 346 (29.2) |
| Class III | 21 (39.6) | 14 (6.7) | 26 (3.3) | 4 (1.3) | 14 (4.7) | 9 (22.5) | 26 (7.7) | 81 (6.8) |
| Class IV | 6 (11.3) |  | 1 (0.1) | 1 (0.3) | 4 (1.3) |  | 3 (0.9) |  |
| Cardiac surgery | 14 (7.8) | 180 (87.8) | 665 (85.1) | 257 (81.1) | 185 (61.7) | 28 (68.3) | 282 (81.3) | 1061 (88.9) |
| Catheter interventions | 12 (6.7) | 122 (58.9) | 202 (25.4) | 117 (36.7) | 209 (70.1) | 39 (97.5) | 140 (39.8) | 462 (38.7) |

IQR: Inter Quartile Range

**Table S2:** Overview of variables and measurements

| Variables | Definition used in this study | Measurements | Interpretation |
| --- | --- | --- | --- |
| Perceived health status | The impact of a disease according to the patient, including symptoms, functional status, and health-related quality of life^1^ | - 12‐item shortened and adapted version of the RAND-36^2^ | - Physical Component Summary (PCS) and Mental Component Summary (MCS) ranging from 0 to 100; - Higher scores = better perceived health |
|  |  | - EuroQol-5D Visual Analog Scale  (EQ-VAS)^3^ | - EQ-VAS score ranging from 0 (worst imaginable health state) to 100 (best imaginable health state); - Higher scores = better perceived health |
| Depressive symptoms | The presence (and frequency) of specified symptoms of depression in the preceding 2 weeks. | Patient Health Questionnaire-8 (PHQ-8)^4^ | - Scores range from 0 to 24. Scores of ≥10 indicate current depression. |
| Anxiety | The presence of symptoms of generalized anxiety | General Anxiety Disorder-7 (GAD-7)^5^ | - Scores range from 0 to 21. Scores of 5, 10, and 15 are taken as cut-off points for mild, moderate and severe anxiety |
| Quality of life | The degree of overall life satisfaction that is positively or negatively influenced by individuals’ perception of certain aspects of life important to them, including matters both related and unrelated to health^6^ | Linear Analog Scale (LAS)^7^ | - LAS score ranging from 0 (worst imaginable quality of life) to 100 (best imaginable quality of life); - Higher scores = better quality of life |

1. Rumsfeld JS, Alexander KP, Goff DC, Jr., et al. Cardiovascular health: the importance of measuring patient-reported health status: a scientific statement from the American Heart Association. *Circulation* 2013; **127**(22): 2233-49.

2. Ware JE, Kosinski M, Turner-Bowker DM, Sundaram M, Gandek B, Maruish ME. User's Manual for the SF-12v2 Health Survey Second Edition. Lincoln, RI: QualityMetric, Incorporated; 2009.

3. EuroQol Group. EuroQol--a new facility for the measurement of health-related quality of life. *Health Policy* 1990; **16**(3): 199-208.

4. Kroenke K, Spitzer Robert L. The PHQ-9: A New Depression Diagnostic and Severity Measure. *Psychiatric Annals* 2002; **32**(9): 509-15.

5. Spitzer RL, Kroenke K, Williams JB, Löwe B. A brief measure for assessing generalized anxiety disorder: the GAD-7. *Arch Intern Med* 2006; **166**(10): 1092-7.

6. Moons P, Van Deyk K, Marquet K, et al. Individual quality of life in adults with congenital heart disease: a paradigm shift. *Eur Heart J* 2005; **26**(3): 298-307.

7. Moons P, Van Deyk K, De Bleser L, et al. Quality of life and health status in adults with congenital heart disease: a direct comparison with healthy counterparts. *Eur J Cardiovasc Prev Rehabil* 2006; **13**(3): 407-13.

**Table S3:** Multivariable general linear mixed models with Gross National Income (GNI) as predictor of PROs, adjusted for patient characteristics (n=8,415)

|  | **PCS** | | **MCS** | | **EQ-VAS** | | **PHQ-8** | | **GAD-7** | | **LAS QOL** | |
| --- | --- | --- | --- | --- | --- | --- | --- | --- | --- | --- | --- | --- |
|  | **Est. (SE)** | **p-value** | **Est. (SE)** | **p-value** | **Est. (SE)** | **p-value** | **Est. (SE)** | **p-value** | **Est. (SE)** | **p-value** | **Est. (SE)** | **p-value** |
| **Gross National Income (Log2)** | 3.0 (0.4) | <0.001 | 0.6 (0.3) | 0.042 | 0.5 (0.3) | 0.147 | 0.02 (0.08) | 0.840 | -0.07 (0.09) | 0.384 | 0.8 (0.3) | 0.016 |
| **Age** | -0.3 (0.02) | <0.001 | -0.01 (0.02) | 0.459 | -0.1 (0.02) | <0.001 | -0.02 (0.005) | <0.001 | -0.04 (0.005) | <0.001 | -0.07 (0.02) | <0.001 |
| **Sex** |  |  |  |  |  |  |  |  |  |  |  |  |
| **Men** | 6.0 (0.4) | <0.001 | 5.9 (0.4) | <0.001 | 2.3 (0.4) | <0.001 | -1.3 (0.1) | <0.001 | -1.4 (0.1) | <0.001 | 1.7 (0.4) | <0.001 |
| **Women** | # |  | # |  | # |  | # |  | # |  | # |  |
| **Employment status** |  |  |  |  |  |  |  |  |  |  |  |  |
| **Part-time or full-time work** | # |  | # |  | # |  | # |  | # |  | # |  |
| **Homemaker or retired** | -7.0 (0.8) | <0.001 | -3.3 (0.0.8) | <0.001 | -2.1 (0.7) | 0.004 | 0.7 (0.2) | <0.001 | 0.3 (0.2) | 0.182 | -2.1 (0.7) | 0.003 |
| **Job seeking, unemployed, or disability** | -15.9 (0.6) | <0.001 | -9.4 (0.6) | <0.001 | -8.4 (0.6) | <0.001 | 1.9 (0.2) | <0.001 | 1.2 (0.2) | <0.001 | -7.9 (0.6) | <0.001 |
| **Full-time student** | -1.3 (0.8) | 0.085 | -1.5 (0.8) | 0.045 | 0.5 (0.7) | 0.525 | 0.2 (0.2) | 0.419 | -0.2 (0.2) | 0.372 | 0.5 (0.7) | 0.442 |
| **Other** | -4.8 (0.8) | <0.001 | -3.6 (0.8) | <0.001 | -2.6 (0.8) | <0.001 | 0.5 (0.2) | 0.008 | 0.2 (0.2) | 0.243 | -1.6 (0.7) | 0.037 |
| **Marital status** |  |  |  |  |  |  |  |  |  |  |  |  |
| **Never married** | -1.2 (0.5) | 0.015 | -1.2 (0.5) | 0.012 | -1.2 (0.5) | 0.011 | 0.3 (0.1) | 0.011 | -0.2 (0.1) | 0.137 | -2.9 (0.5) | <0.001 |
| **Married or living with partner** | # |  | # |  | # |  | # |  | # |  | # |  |
| **Divorced or widowed** | -3.6 (1.0) | <0.001 | -5.6 (1.0) | <0.001 | -3.1 (0.9) | <0.001 | 1.5 (0.2) | <0.001 | 0.9 (0.3) | <0.001 | -5.0 (0.9) | <0.001 |
| **Other** | 9.0 (3.7) | 0.014 | 7.2 (3.7) | 0.049 | 2.6 (3.5) | 0.453 | -0.4 (0.9) | 0.665 | -1.2 (1.0) | 0.211 | 3.0 (3.4) | 0.380 |
| **Disease complexity** |  |  |  |  |  |  |  |  |  |  |  |  |
| **Simple** | 2.7 (0.8) | <0.001 | 1.4 (0.8) | 0.067 | 0.8 (0.7) | 0.244 | -0.2 (0.2) | 0.213 | -0.09 (0.2) | 0.665 | 0.6 (0.7) | 0.373 |
| **Moderate** | 1.9 (0.5) | <0.001 | 0.8 (0.5) | 0.107 | -0.2 (0.5) | 0.730 | 0.08 (0.1) | 0.520 | 0.1 (0.1) | 0.438 | 0.4 (0.5) | 0.379 |
| **Complex** | # |  | # |  | # |  | # |  | # |  | # |  |
| **Physiological stage** |  |  |  |  |  |  |  |  |  |  |  |  |
| **Stage A** | 17.7 (1.0) | <0.001 | 8.8 (1.0) | <0.001 | 11.2 (1.0 | <0.001 | -1.7 (0.3) | <0.001 | -1.1 (0.3) | <0.001 | 8.8 (1.0) | <0.001 |
| **Stage B** | 15.3 (0.9) | <0.001 | 6.2 (0.9) | <0.001 | 9.4 (0.8) | <0.001 | -1.1 (0.2) | <0.001 | -0.7 (0.2) | 0.002 | 6.5 (0.8) | <0.001 |
| **Stage C** | 11.9 (0.8) | <0.001 | 5.2 (0.8) | <0.001 | 7.5 (0.8) | <0.001 | -0.9 (0.2) | <0.001 | -0.6 (0.2) | 0.006 | 5.6 (0.8) | <0.001 |
| **Stage D** | # |  | # |  | # |  | # |  | # |  | # |  |
| **History of cardiac surgery** | 1.7 (0.5) | 0.001 | 0.9 (0.5) | 0.082 | 0.2 (0.5) | 0.690 | -0.05 (0.1) | 0.721 | -0.07 (0.1) | 0.580 | -0.4 (0.5) | 0.387 |
| **History of catheter interventions** | 2.2 (0.5) | <0.001 | 1.7 (0.4) | <0.001 | 1.6 (0.4) | <0.001 | -0.3 (0.1) | 0.01 | -0.2 (0.1) | 0.116 | 1.5 (0.4) | <0.001 |
|  |  |  |  |  |  |  |  |  |  |  |  |  |
| **Center as random effect** | * |  | * |  | * |  | * |  | * |  | * |  |

# = reference category; Est.=Estimates; SE=Standard Error; PCS=Physical Component Summary; MCS=Mental Component Summary; EQ-VAS=EuroQol 5 Dimensions-Visual Analog Scale; PHQ-8= Patient Health Questionnaire-8; GAD-7= General Anxiety Disorder-7; LAS QOL= Linear Analog Scale Quality of Life
